# Notices and Reviews

**Published:** 1890-06

**Authors:** 


### Book Notices.

THE PHYSICIANS VISITING LIST. Philadelphia, P. Blakiston, Son & Co.

This is one of the best visiting lists published. It has been in existence thirty-eight years B.

NERVOUS SYPHILIS, by H. C. Wood, M. D., LL. D., of Philadelphia. Geo. S. Davis, Publisher, Detroit, Mich.

SOME FALLICIES CONCERNING SYPHILIS, by E. L. Keyes, M. D., New York. Geo. S. Davis, Publisher, Detroit, Mich.

These two little books belong to the Leisure Library series and are valuable productions. Twenty-five cents each.

MEDICAL AND SURGICAL MEMOIRS—Containing investigations on Geographical Distribution, Causes, Nature, Relation and Treatment of Various Diseases, from 1855 to 1890. By Joseph Jones, M. D., Professor of Chemistry and Clinical Medicine in Medcal Department of Tulane University of Louisiana at New Orleans. Vol. III., in two parts. Illustrated by eight chromolithographic plates, twenty-one maps and charts, twenty-two extensive and elaborate tables and seventeen engravings. New Orleans, La.: Joseph Jones, M. D., 150 Washington Avenue, corner Camp street, Fourth District. 1890. All rights reserved.

Parts I. and II. of vol. III. of Prof. Jones' Memoirs are before the reviewer. These parts contain some 800 or 1000 pages each, besides a great number of useful maps, charts and tables. A number of fine colored plates are to found in these books. The Medical and Surgical Memoirs of Prof. Jones is a miscellaneous work of very great value. It contains the best information on all public matters relating to hygiene and sanitation, and gives a history and the practical operation of quarantine laws during the epidemics in New Orleans and elsewhere. The work contains a good deal of original investigation; in fact, embraced therein is the entire life-work of the author on all subjects of scientific character during an active professional career of over thirty-five years. Prof. Jones is one of the hardest workers and most painstaking writers of the age. This work should be in the library of every well regulated physician for reference and study. B.

---

OPHTHALMOLOGY AND OPHTHALMOSCOPY for Practitioners and Students of Medicine, by Hermann Schmidt-Rimpler, Professor of Ophthalmology and director of the Ophthalmological Clinic in Marburg. Translated from the third German revised edition, edited by D. B. St. John-Roosa, M. D., LL. D., New York. 183 wood cuts and three colored plates, pp. 564, New York: Wm. Wood & Co., 1889.

This is a standard work well suited to the general practitioner as well as to the specialist. The fact that St. John-Roosa has had the editing of this edition is a sufficient guarantee to the American profession. Schmidt-Rimpler has been well and favorably known a long time. The publishers, Wm. Wood & Co., seldom touch anything but the best. The book, altogether, is what the profession has a right to expect, coming from such source.

The chapter on errors of refraction is specially comprehensive.

B.

---

A TEXT-BOOK OF PRACTICAL MEDICINE, designed for the use of Students and Practitioners of Medicine. By Alfred L. Loomis, M. D., LL. D., Professor of Pathology and Practical Medicine in the Medical Department of the University of the City of New York, Visiting Physician to the Bellevue Hospital, etc. Eighth edition. Revised and enlarged, with 215 illustrations. New Yoik, Wm. Wood & Co., 1889.

This work has been thoroughly revised and rewritten. About fifty pages have been added, making in all 1174 pages. Many important changes have been made. Among these aie "brief descriptions of the more frequent pathological processes and detailed statements of the methods employed in bacteriological study, with an enumeration of the distinguishing characteristics of those micro-organisms which at the present time are regarded as pathogenic. Several additions have been made to the list of diseases considered." In fact, the book is up to date in every respect. It enjoys the distinction of being a standard work, which is almost enough to say of any production. In the opinion of the writer, Loomis' Practice is one of the best published.

B.

---

A TEXT-BOOK OF ANIMAL PHYSIOLOGY, with introductory chapters on General Biology and a full treatment of Reproduction, for students of Human and Comparative (veterinary) Medicine and of General Biology. By Wesley Mills, A. M., M. D., L. R. C. P. (Eng.), Prof. of Physiology in McGill University and the Veterinary College, Montreal. Over 500 illustrations. New York: D. Appleton & Co.; pp. 690. 1889.

It can be said of this work that it is original in so far as it discusses the subjects of human and animal physiology jointly, making the work suitable alike for medical and veterinary students. It is really a scientific production, and indispensable for reference and study to teachers of physiology as well as to veterinary surgeons and medical men. The book deserves a large sale. B.

